# Carbon footprint of a sample of clinical trials for people with neurological disorders: cross-sectional analysis

**DOI:** 10.1136/bmjopen-2024-090419

**Published:** 2025-06-17

**Authors:** Denise Cranley, Sarah Dunn, John-Paul Taylor, Michael JR Desborough, Jennifer Craig, Nikola Sprigg, Rachel McComish, Thomas Foltynie, Joanna Wardlaw, Katherine Oatey, Anna Heye, Philip Bath, Karen Innes, Lynn Dinsmore, Jessica Griffiths, Lisa Fox, Paula R Williamson, Rustam Al-Shahi Salman

**Affiliations:** 1 Edinburgh Clinical Trials Unit, The University of Edinburgh, Edinburgh, UK; 2 Newcastle Clinical Trials Unit, Newcastle University, Newcastle upon Tyne, UK; 3 Institute of Neuroscience, Newcastle University, Newcastle upon Tyne, UK; 4 NHS Blood and Transplant, John Radcliffe Hospital, Oxford, Oxfordshire, UK; 5 Oxford Clinical Research in Transfusion Medicine, Univeristy of Oxford, Oxford, Oxfordshire, UK; 6 Mental Health & Clinical Neuroscience, University of Nottingham, Nottingham, UK; 7 Division of Stroke Medicine, University of Nottingham, Nottingham, UK; 8 Comprehensive Clinical Trials Unit, University College London, London, UK; 9 Institute of Neurology, University College London, London, UK; 10 Centre for Clinical Brain Sciences & Brain Research Imaging Centre, University of Edinburgh, Edinburgh, UK; 11 Institute of Cancer Research Clinical Trials and Statistics Unit (ICR-CTSU), London, UK; 12 Biostatistics, University of Liverpool, Liverpool, UK; 13 Centre for Clinical Brain Sciences, The University of Edinburgh, Edinburgh, Scotland, UK

**Keywords:** Clinical Trial, NEUROLOGY, Climate Change

## Abstract

**Abstract:**

**Objective:**

To quantify the carbon footprint of a sample of clinical trials for neurological disorders.

**Design:**

Cross-sectional study.

**Method:**

Two clinical trial registries were searched on 29 December 2022 for phase 2–4 randomised controlled trials led from and recruiting in the UK, enrolling people with any of the 15 neurological disorders with the highest global burden, that had started recruitment or been registered in the preceding 5 years. Eligible trials were invited to share data to estimate emissions in each of the 10 modules of the Low Carbon Clinical Trials footprinting guidance. The primary outcome measure was kg of carbon dioxide equivalent (CO_2_e).

**Results:**

318 randomised controlled trials were found, nine were eligible and six shared data (three completed and three ongoing). The module with the highest estimated CO_2_e for each trial was the Clinical Trial Unit staff emissions (median 24 126 kg CO2e, IQR 10 395–78,867; range 45–79% of overall emissions of each trial); commuting accounted for >50% of CO_2_e in this module. The second and third highest modules were trial-specific participant assessments (median 11 497 kg CO2e, IQR 825–15,682) and trial supplies and equipment (median 1161 kg CO_2_e, IQR 226–6632). The total carbon footprint of these six trials involving 2248 participants at 239 sites was 2 63 215 kg CO_2_e.

**Conclusions:**

Emissions by Clinical Trials Unit staff were the top modifiable carbon hotspot in six randomised controlled trials for people with neurological disorders, which had a total carbon footprint equivalent to 1364 passengers’ return aeroplane journeys between London and Edinburgh.

**STRENGTHS AND LIMITATIONS OF THIS STUDY**

- This study used a standardised approach to estimate the carbon footprint of a sample of randomised controlled trials for people with neurological disorders.
- We included a modest number of randomised controlled trials and neurological disorders.
- The estimated carbon footprint of the ongoing trials may vary after closure due to subsequent changes in the protocol.
- This study was unable to capture the specific effects of COVID-19 on the carbon footprint of the included randomised controlled trials.

## Introduction

 Climate change is one of the most significant global threats to health and economies.^1 2^ Clinical trials are integral to routine healthcare; however, they make a significant contribution to greenhouse gas emissions. Adshead *et al* estimated the carbon release of all 350 000 clinical trials registered with the Clinicaltrials.gov database was 27.5 million tonnes.^3^ Funding agencies and other stakeholders in clinical trials are responding with guidelines and workshops to implement carbon reduction strategies.^4 5^

Studies of the carbon footprint of clinical trials are scarce but include: 12 trials funded by the National Institute for Health and Care Research Health Technology Assessment 2002–2003, using a complex carbon conversion factor formula^6^; two trials of drugs for head injury or traumatic bleeding, using a carbon audit company^7^; one trial of nocturnal haemodialysis versus standard care, using an online carbon calculator^8^; three trials funded by AstraZeneca using an in-house carbon calculator tool^9^; eight industry-sponsored phase 1– phase 4 trials^10 11^ and a number of clinical trials that used the Low Carbon Clinical Trials Group guidance, which was used in this study.^12 13^ These studies concluded that trial team travel, emissions from study sites and participant travel for assessments are the key contributors to the carbon footprint of clinical trials.

Until recently, specific tools have not existed to measure clinical trials’ carbon footprints comprehensively, reliably and consistently and to measure the effects of interventions to reduce emissions. Therefore, in 2021, the Low Carbon Clinical Trials Group proposed a strategy to decarbonise clinical trials^3^ and subsequently developed the National Institute for Health Research (NIHR)-funded ‘Detailed guidance and method to calculate the carbon footprint of a clinical trial’.^12^ The guidance is designed specifically for academic (ie, investigator-initiated and non-commercially funded) clinical trials to quantify the carbon emissions of trial-specific activities above and beyond standard of care in 10 modules covering trial set up, conduct and closure (table 1), which they piloted with three clinical trials.^12 13^

**Table 1:** Modules in the Low Carbon Clinical Trials carbon footprinting guidance tool

Module	Exemplar components
Trial set up	Preparation and shipment of paper investigator site files
Clinical Trials Unit emissions	Commuting and facilities energy used by staff
Trial-specific meetings and travel	Travel to meetings, videoconferencing, overnight stays and subsistence
Trial intervention	Shipment of investigational medicinal products or devices
Data collection and exchange	Email exchange, return of paperwork from patients or site teams
Trial supplies and equipment	Data linkage and equipment used by patients
Trial-specific patient assessments	Study visits, scans, blood samples and the energy consumption of site staff
Samples	Return of samples to the laboratory. Guidance also includes shipment of sample kits and sample kit materials
Laboratory	Storage and analysis of samples and energy consumption of laboratory staff
Analysis and trial close-out	Archiving of paperwork and electronic data

Until now, we are not aware of studies of the carbon footprint of clinical trials for neurological disorders, which were the leading cause of disability-adjusted life years in 2021.^14^ The carbon emissions of clinical trials for neurological disorders are likely to be significant because the disease burden provokes clinical trial activity, and they use a wide range of drugs, devices and outcome measures including brain imaging.^15^,^17^

Therefore, we set out to apply the Low Carbon Clinical Trials Group’s carbon footprinting guidance to a sample of clinical trials focused on neurological disorders. Our objectives were to determine which modules and components of these clinical trials account for the highest proportions of their carbon emissions, and whether these aspects would be amenable to change.

## Methods

We conducted a cross-sectional observational study to estimate the carbon emissions of a sample of clinical trials for neurological disorders in the UK retrospectively.

We sought to include phase 2–4 randomised controlled trials (RCTs) (in set up, recruiting, in follow-up or completed), led from and recruiting in the UK, enrolling people with any of the 15 neurological disorders with the highest global burden (stroke, migraine, dementia, meningitis, epilepsy, spinal cord injury, traumatic brain injury, brain cancer, tension type headache, encephalitis, Parkinson’s disease, tetanus, multiple sclerosis, motor neuron disease and other neurological conditions)^18^ and had started recruitment or been registered in the 5 years before the search date. RCTs were not eligible if their chief investigator was unwilling or unable to share data, or there was no trial protocol published in a trial register, journal or website (see Preferred Reporting Items for Systematic Reviews and Meta-Analyses (PRISMA) diagram, online supplemental appendix C, in the supplementary file).

On 29 December 2022, one author (DC) searched for eligible RCTs in Clinicaltrials.gov and the WHO International Clinical Trials Registry Platform (WHO ICTRP).^19^ RCTs were included if they were registered within the previous 5 years to ensure the results are relevant to current practice and to ensure we reflected the carbon footprint of contemporary RCT designs. The electronic searches included text words for the 15 neurological disorders with the highest global burden,^18^ and other terms reflecting the eligibility criteria (see online supplementary file, online supplemental appendix B). One author (DC) de-duplicated and exported records from Clinicaltrials.gov and WHO ICTRP, screened them against the study's eligibility criteria (se online supplemental Appendix A) and resolved any uncertainties in discussion with the senior author (RA-SS).

We extracted data about the included RCTs from the trial registry or their protocols. We sent a bespoke source data collection sheet to each RCT team to capture the components of each module of the footprinting guidance (table 1) and resolved any uncertainties with RCT teams via a Microsoft Teams meeting.

We developed a Microsoft Excel worksheet to collate our calculations and to record any assumptions made for reproducibility and transparency. We followed the guidelines, emission factors and formulae in the NIHR-funded detailed guidance and method to calculate the carbon footprint of a clinical trial developed by the Institute of Cancer Research and University of Liverpool, on behalf of the Low Carbon Clinical Trials Group.^12^ We used V.0.1 18 January 2023 of the guidance; subsequent versions of the guidance include additional calculations and emission factors.^12^ We applied the guidance to the included RCTs retrospectively because three clinical trials had completed recruitment and follow-up at the time of this study, but the other three RCTs remained active. Access to specific details was limited for some of the completed RCTs, requiring us to use estimates instead (eg, weight of some shipments, distances between sites, type of packaging material used for shipment and number of questionnaires returned). We made some assumptions about active RCTs to permit estimations of their overall carbon footprint (eg, the duration of the study was estimated from the protocol, numbers of boxes for archiving were estimated in discussion with the RCT team and the target sample size stated in the protocol was used for calculations).

The primary outcome measure was kilograms of carbon dioxide equivalent (kg CO_2_e), which is the unit of measurement for the warming effect of greenhouse gases; we also expressed this in metric tonnes. Emissions of greenhouse gases other than carbon are quantified using an exchange rate, which expresses how many kilograms of CO_2_ emissions warm the climate as much as 1 kg of another greenhouse gas. We quantified kg CO_2_e for each of the 10 modules and each subsection or component of each module for each included RCT. Our main aim was to compare the relative contributions of the modules of each RCT to their total carbon footprint, rather than their absolute kgCO_2_e, given the variation in geographical scope, interventions and sample size between the included RCTs.

## Results

We found 318 records of clinical trials for neurological disorders (198 from Clinicaltrials.gov and 120 from WHO ICTRP), of which nine were eligible for this study, covering four neurological conditions (ischaemic stroke, intracerebral haemorrhage, dementia or Parkinson’s disease). The PRISMA flow diagram outlines the selection process and is available in the online supplemental Appendix C. We approached the chief investigator and trial manager of the nine eligible RCTs; the chief investigators of seven RCTs agreed to participate, and six RCTs (67%) shared data.

The COmBining memantine And cholinesterase inhibitors in Lewy body dementia Treatment (COBALT) trial aimed to determine the clinical and cost effectiveness of 12 months of therapy with up to 20 mg daily oral memantine in addition to acetylcholinesterase inhibitor therapy in people with dementia with Lewy bodies (COBALT-DLB) and Parkinson’s disease dementia (COBALT-PDD) (ISRCTN79794378).^20^ The Desmopressin for reversal of Antiplatelet drugs in Stroke due to Haemorrhage (DASH) trial aimed to determine the feasibility of a definitive RCT of the safety and efficacy of desmopressin for patients with antiplatelet-associated intracerebral haemorrhage (ICH) (ISRCTN67038373).^21^ The Exenatide-PD3 study aimed to determine the effects of exenatide once weekly over 2 years as a potential disease-modifying treatment for Parkinson’s disease; at the time of this study, it was in follow-up and recruitment was closed (ISRCTN14552789).^22^ The LACunar Intervention Trial-2 aimed to estimate the safety and efficacy of cilostazol and isosorbide mononitrate in a phase IIb 2×2 factorial trial to determine whether a definitive RCT would be feasible in the UK (ISRCTN14911850).^23 24^ The Pharyngeal Electrical stimulation for Acute dysphagia Trial (PhEAST) aimed to assess whether pharyngeal electrical stimulation is safe and effective in improving dysphagia after stroke (ISRCTN98886991).^25^ The REstart or STop Antithrombotics Randomised Trial (RESTART) after stroke due to intracerebral haemorrhage aimed to estimate the safety of starting versus avoiding antiplatelet therapy (ISRCTN71907627).^26^ A more detailed breakdown of the results per RCT is available in online supplemental figure 12-18, online supplemental appendix E.

Three of the included RCTs had completed, and the other three were active at the time of data collection (see Table 2 in online supplemental appendix F, supplementary file). The RCTs varied in duration (3–8 years), numbers of full-time equivalent (FTE) staff in the team (2-12), sample size (54-800), number of sites (6-112), trial type (5/6 were Clinical Trials of Investigational Medicinal Products [CTIMPs]), brain imaging (three performed scans in addition to standard care), number of research visits per participant (0–10), collection of additional samples (2/6 studies collected samples) and duration of archiving (5–10 years), culminating in a range of estimated total carbon emissions for each of the six RCTs between 8805 and 1 06 429 kg CO_2_e. In total, the six RCTs included 239 sites and 2248 participants (954 recruited and 1294 planned).

The module with the highest estimated CO_2_e was Clinical Trial Unit (CTU) emissions (median 24 126 kg CO_2_e, IQR 10 395–78,867), within which the commute to the CTU accounted for >50% of the total CO_2_e in this module for each RCT (online supplemental figure 2 and 3). The module with the second highest estimated CO_2_e was trial-specific participant assessments (median 11 497 kg CO2e, IQR 825–15,682) (online supplemental figure 8). The module with the third highest estimated CO_2_e was trial supplies and equipment (median 1161 kg CO_2_e, IQR 226–6632) (online supplemental figure 7). The data collection and exchange module had a median footprint of 845 kg CO_2_e (IQR 317–2065) (online supplemental figure 6). The data analysis and archiving module had a median footprint of 563 kg CO_2_e (IQR 158–1616) (online supplemental figure 11). The trial specific meetings module had a median footprint of 328 kg CO_2_e (IQR 157–1588) (online supplemental figure 4) and the trial set up module (online supplemental figure 1) had a median footprint of 249 kg CO_2_e (IQR 41–873). The treatment intervention module (online supplemental figure 5) had a median footprint of 9 kg CO_2_e (IQR 0–126), the laboratory emissions module (online supplemental figure 10) had a median footprint of 0 kg CO_2_e (IQR 0–71) and the sample analysis module (online supplemental figure 9) had a median footprint of 0 kg CO_2_e (IQR 0–51). See online supplemental material, Appendix D and E (online supplemental figure 1-11), for a full breakdown of the estimated carbon emissions for each module and each RCT. The estimated total carbon footprint for the six included clinical trials was 2 63 215 kg CO_2_e (see Table 2 in online supplemental appendix F, supplementary file).

Comparing the relative contributions of each module within the RCTs (see online supplemental appendix G, table 3 in online supplemental file), trial set up, trial specific meetings, treatment interventions, samples and laboratory emissions accounted for similarly small proportions in all RCTs. CTU emissions were consistently the highest proportion, although they varied from 45–79% of overall emissions. Some trials had especially high proportions of emissions due to data collection, trial supplies, patient assessments and archiving due to the frequency of data collection, nature of the intervention, number of patient visits and duration of archiving (table 1).

## Discussion

### Summary of findings

We estimated the total carbon footprint for six RCTs for neurological disorders to be 2 63 461 kg CO_2_e. This is equivalent to 295 116 pounds of coal burned or 25 880 gallons of diesel consumed.^27^ It is also the equivalent to return plane journeys between London and Edinburgh for 1364 passengers.^28^ The proportions of CO_2_e in six RCTs for people with neurological disorders were likely to be influenced by the individual characteristics of each RCT, including the duration and FTE of CTU staff, the number of study visits, the inclusion of scans, the number and format of trial-specific meetings, the number of samples collected, temperature requirements for shipments and the duration and amount of archiving required.

CTU emissions were consistently the highest proportion of CO_2_e, with the trial team commute accounting for 62–75% of this module in each RCT. Trial-specific patient assessments accounted for the second highest proportion of CO_2_e for five out of six RCTs. The sixth RCT (PhEAST) did not have additional study assessments or scans, beyond routine care. Videoconferencing has a low emission factor, and the use of this technology contributed to a lower carbon footprint for the RCTs that used remote/hybrid meetings. Additionally, for RCTs that required temperature-controlled shipments, this emission factor contributed to a higher carbon footprint for that RCT than an intervention which did not require temperature control (the emissions associated with the manufacture of the investigational medicinal product (IMP) were beyond the scope of this tool and were not included). Two of the RCTs used IMP that was prescribed locally, which also contributed to a lower carbon footprint for those RCTs. Sample analysis and laboratory staff had low contributions for these six RCTs as four of the RCTs did not collect samples. One RCT had samples shipped on dry ice and stored in a −80°C freezer which contributed to the overall carbon footprint.

The findings of this study support previous research carried out in this area. Lyle *et al*^6^ concluded that trial team travel and emissions from RCT sites and participant-related travel had the highest emissions on average. Subaiya *et al*^7^ found the trial team emissions in the Clinical Randomisation of an Antifibrinolytic in Significant Haemorrhage 2 (CRASH-2) trial were the highest contribution. Quann *et al*^8^ also found that carbon emission savings can be made in relation to trial travel and visits, reduced office commutes and the use of technology for study training, meetings and study visits. MacKillop *et al*^9^ highlighted some areas of intense carbon emissions in their three international RCTs, including travel for meetings and the sample life-cycle from sample kits, shipment and storage.^9^ The Low Carbon Clinical Trials Group applied the guidance to three RCTs and found that their carbon footprint was largely attributable to CTU emissions and staff travel and trial-specific in-person participant assessments.^12 13^ Others reported similar findings with a range of industry-sponsored phase 1 to phase 4 trials and found hotspots with drug manufacture, patient and staff travel and sample handling.^10 11^

### Strengths and limitations

This study has successfully estimated and identified the carbon hotspots of six RCTs of neurological disorders, and it is only the second study to evaluate the carbon footprint for research into neurological disorders since the CRASH trials. We were able to include two-thirds of the eligible clinical trials, but the generalisability of the carbon footprints of these six RCTs about dementia, movement disorders and stroke to all neurological disorders is uncertain. However, the RCTs’ characteristics were similar to a recent review of neurology trial registrations,^29^ and taking stroke due to ICH as an example, the characteristics of RESTART and DASH were similar to those included in a systematic review of all clinical trials for this condition.^30^ Similarly, this study included both drug and a device trial, with a range of trial outcomes. The study benefitted from using a standardised approach to estimate the carbon footprint of clinical trials using the recently published NIHR-funded ‘Detailed Guidance and method to calculate the carbon footprint of a clinical trial’.^12^

However, only a small number of RCTs were eligible and included in this study, and the six RCT designs were heterogeneous. It is also acknowledged that the estimated total carbon footprint for some of the six RCTs on close-out may be higher or lower than calculated here. These calculations were based on study protocols up to March 2023 (e.g. study duration, number of participants or study visits), and these RCTs may amend their protocols before completion. Furthermore, the inclusion criterion of starting recruitment or being registered in the 5 yearsbefore the search date gincluded the disruption to RCTs during the COVID-19 pandemic, and we acknowledge that this may have contributed to a reduction in estimated CO_2_e within some of the RCTs due to a change in practice; however, these data were not collected due to added complexities of carbon calculations. Finally, not all data were available for some of the RCTs as the study team was unable to predict some modules, for example, number of samples, number of archiving boxes, number of follow-up questionnaires and storage size (gigabytes) of a trial database. So in some instances, these modules may have been underestimated due to unavailable data (see online supplemental appendix E in supplementary material for full details). Additionally, we have not accounted for multipurpose meetings and trips, and there may be efficiency gains from travelling to some meetings that serve multiple purposes. Ideally, the carbon calculator tool will be used prospectively during the study design stage to inform protocol authors about predicted carbon hotspots. It should be noted that the guidance only accounts for the carbon footprint of a RCT; water use, land use, social and economic impacts are not currently captured.^5^

### Recommendations for future research

Future research should enlarge our sample size and study a broader selection of RCTs for neurological disorders. The NIHR-funded guidance and method should also be applied prospectively to RCTs from trial design to completion. Additionally, future examination of trial protocol adaptation as a result of COVID-19 could provide important insights into which factors might drive carbon footprint changes. Most importantly, future research should test interventions in RCTs to reduce CO_2_e, for example, as studies within a trial (SWATs) within individual RCTs or as cluster RCT SWATs where RCTs are allocated to CO_2_e reducing interventions.

### Recommendations for future trial design

This study also highlights where sustainable practices could be implemented. Trial teams should consider maximising the use of technology when designing their study and introduce remote assessments, remote consent and remote meetings where possible. Preference should be given to using existing technology and hardware to minimise the carbon footprint of trial equipment; for example, avoiding the provision of new smartphones unless absolutely necessary. Participants in the Nightlife RCT^8^ were supportive of remote assessments, and implementation of remote study visits, meetings and training can improve diversity within the trial as the trial becomes more accessible, although consideration needs to be given to the potential for digital exclusion.

Videoconferencing is not carbon neutral, and it does produce a carbon footprint, as can be seen in all of the RCTs (online supplemental figure 4), but the impact is minimal and drastically reduces the higher impact of travel for meetings. Further solutions aimed at reducing the impact of videoconferencing include considering the necessity of meetings, the number of people joining/streaming the call and perhaps even the necessity of keeping a video on during a meeting.

Supporting departments involved in trial assessments such as scanning and sample processing can also consider their role in reducing the carbon footprint of RCTs. Imaging departments should consider suitable actions to reduce the carbon emissions for imaging in research; for example, MRIs have a higher carbon footprint than CTs, and a recent study suggested switching the power on MRIs from idle to off mode for 12 hours overnight can save 8.7–14.9 tonnes of CO_2_e.^17^ Consideration should also be given to sample collection and advice from frameworks such as My Green Lab when designing a study. Researchers can ensure only required samples are collected and stored for the minimum time necessary. It may also be possible to examine whether study samples can be stored in a −70°C freezer instead of a −80°C without adversely affecting the quality of samples. Health economists may also consider the carbon footprint of clinical trials in the context of the extent to which their results affect the carbon footprint of clinical practice.

### Recommendations for stakeholders

Research institutions should consider the development of greener infrastructure when planning buildings and commuting links. They should also introduce policies and incentives for sustainable staff travel for daily commutes, conferences and meetings.

Governing bodies such as competent authorities and sponsors should consider how to use and implement electronic monitoring, safety and oversight processes for clinical trials. Additionally, streamlining electronic trial data processes could reduce trial paperwork and reduce long-term archiving of data, as well as encouraging the use of electronic consent options and remote study visits where possible.

Research funding bodies should consider introducing greener requirements and incentives when applying for funding.^3^

## Conclusion

This study has highlighted the carbon hotspots for six RCTs for people with neurological disorders in the UK. These hotspots include CTU emissions and trial team commutes, patient visits and assessments, new equipment, archiving and the use of scans and refrigerated IMP deliveries as the main contributors. Areas where we can reduce carbon footprints of clinical trials include the awareness and development of sustainable travel practices by clinical trial staff, an increased use of technology and a change in practice where possible from funders and governing bodies to encourage more sustainable research activity for staff and participants.

## Supplementary material

10.1136/bmjopen-2024-090419 online supplemental file 1

## Data Availability

All data relevant to the study are included in the article or uploaded as supplementary information.
