# Supplementary material for: Carbon footprint of a sample of clinical trials for people with neurological disorders: cross-sectional analysis
Source: BMJ Open. 2025 Jun 17;15(6):e090419. doi: 10.1136/bmjopen-2024-090419 (PMC12182044; doi:10.1136/bmjopen-2024-090419)
Supplement: online supplemental file 1 [file bmjopen-15-6-s001.docx]

**Supplementary Material**

# Appendix A: Inclusion/Exclusion Criteria

**Inclusion Criteria**

(i) Randomised controlled trials.

(ii) Studies which have UK sites. (The tool is based on UK carbon costs)

(iii) Participants must be patients diagnosed with a neurological condition. Specifically, this will be limited to one of the top 15 neurological health conditions, based on the incidence of disability and death in the GBD 2016.

(iv) No age restrictions.

(v) Studies status: Studies can be active not recruiting, recruiting or completed and will include studies with or without results.

(vi) Timeline: RCTs which have (i) started recruitment or (ii) been registered (depending on the trial registry) in the last 5 years.

(vii) Phase 2, 3 and 4 studies only.

**Exclusion Criteria**

(i) No protocol available via a journal, website or trial register.

(ii) Studies that are unwilling or unable to share data.

# Appendix B: Search terms used in the trial registry databases

| Clinicaltrials.gov Advanced Search Terms: | WHOICTRP Advanced Search: |
| --- | --- |
| Condition/disease: Name of each neurological disorder  Other term: Blank  Study Type: Interventional Studies (Clinical Trials)  Study Results: All Studies with or without results  Status:  Recruitment: (i) Recruiting (ii) Active, not recruiting (iii) Completed  Expanded Access: No options selected  Eligibility Criteria:  Age or Age Group: No options selected  Sex: All  Accepts Healthy Volunteers: No option selected  Targeted Search:  Intervention/treatment: Blank  Title/Acronym: Blank  Outcome Measure: Blank  Sponsor/Collaborator: Blank  Sponsor (Lead): Blank  Study ID’s: Blank  Locations:  Country: United Kingdom  City & Distance: Blank  Location Terms: Blank  Additional Criteria:  Phase: Phase 2 and Phase 3 and Phase 4 selected  Funder Type: No options selected  Study Documents: No option selected to avoid the search results being too restrictive. It is also possible to find a study protocol from sources other than this trial registry. However this box will be selected if the search results yield a very high number of studies.  FDAAA 801 Violations: No option selected  Results Submitted: Blank  Study Start: From 21/11/2017 (5 years) To Date of Search  Primary Completion: Blank  First Posted: From 21/11/2017 (5 years) To Date of Search  Last Update Posted: Blank | Title: Name of each neurological disorder  Condition: Left blank  Intervention: Blank  Restrict to COVID-19: No option selected  Search for clinical trials in children: No option selected  Recruitment status: All  Primary sponsor: Blank  Secondary ID: Blank  Country of recruitment: United Kingdom  Date of Registration: 21/11/2017 and 21/11/2022  Phases: Phase 2 and Phase 3 and Phase 4  With results only: No option selected  Rare diseases/orphan drugs: No option selected  Genome editing: No option selected |

# Appendix C: PRISMA Diagram

(See Appendix A for details of the inclusion/exclusion criteria)

**Identification of studies via trial registers**

Records identified from:

Clinicaltrials.gov

(n = 198)

Records identified from:

WHOICTRP

(n = 120)

**Identification**

Records after duplicates removed

(n = 114)

Records after duplicates removed

(n = 161)

Records assessed for eligibility

(n = 161)

Records assessed for eligibility

(n = 114 )

**Screening**

Records excluded:

Study type/non-RCT (n =0)

Non-UK (n = 0)

Non-neuro (n = 5)

Study Status (n = 0)

Phase 1 (n = 0)

UK Sponsor (n = 0)

No protocol available (n = 90)

Unknown (n=60)

Records excluded:

Study type/non-RCT (n =3 )

Non-UK (n = 0)

Non-neuro (n = 7 )

Study Status (n = 43)

Phase 1 (n = 8)

UK Sponsor (n = 37)

No protocol available (n = 13)

Studies included in review

(n = 6)

Studies included in review

(n = 3 )

**Included**

Total studies

(n = 9)

Declined to participate: n = 2

Withdrew due to a delay with non-disclosure agreement = 1

**Participated: n = 6**

# Appendix D: Detailed breakdown of the estimated carbon emissions per module (1 – 10)

| Key |  |
| --- | --- |
| N/A = Not applicable | This is used when a module does not apply to the trial, usually because the trial design meant it was not needed or the trial team did not use it. For example, a trial may not have collected any samples so that module does not apply. Or for some trials, a hotel stay for a meeting is N/A because they did not stay overnight. |
| Unk = Unknown | This is used when the study team were unable to provide data based on predictions. This usually applied to studies which were still active. For example, some studies were unable to predict what the GB storage for the study database will be. |

#### Module 1, Trial Set Up

Module 1 (Figure 1) of the carbon calculator tool estimates the emissions per trial for trial set up. This includes components such as preparation and shipment of the paper Investigator Site File (ISF). Information on number of sites used for individual studies can be found in the Table of Characteristics (Table 2).

The total contribution from the six RCT’s to the carbon emissions of this module is 2,458 kgCO_2_e.


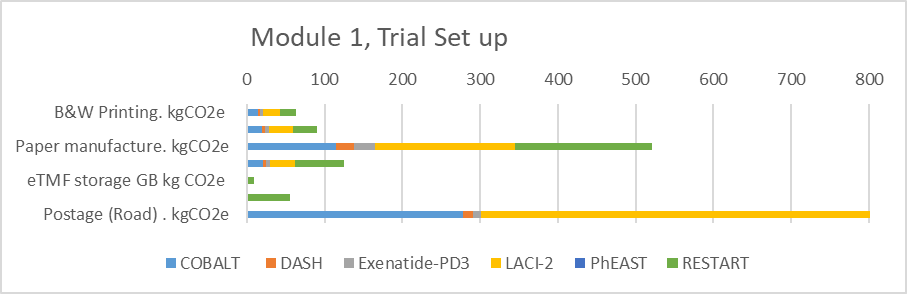


|  | B&W Printing. kgCO2e | Colour printing. kgCO2e | Paper manufacture. kgCO2e | Folder manufacture. kgCO2e | eTMF storage GB kg CO2e | Postage (Air). kgCO2e | Postage (Road) . kgCO2e |
| --- | --- | --- | --- | --- | --- | --- | --- |
| COBALT | 14.00 | 19.90 | 114.90 | 20.50 | 0.12 | N/A | 277.70 |
| DASH | 2.80 | 3.90 | 22.90 | 4.10 | 0.16 | N/A | 13.40 |
| Exenatide-PD3 | 3.40 | 4.80 | 27.50 | 4.90 | 0.80 | N/A | 10.10 |
| LACI-2 | 21.87 | 31.00 | 179.20 | 32.00 | 0.32 | N/A | 534.90 |
| PhEAST | Unk | Unk | Unk | Unk | 0.03 | N/A | Unk |
| RESTART | 21.54 | 30.51 | 176.45 | 63.00 | 7.87 | 55.80 | 737.20 |

Supplementary Figure 1 Estimated sub components of module 1 for all RCT’s

#### Module 2, CTU Emissions

Module 2 (Figure 2) estimates the emissions for the trial team and within that there are three components: Electricity consumption, heat consumption and commute to the office. This is calculated based on the FTE of the trial team which are listed in the Table of Characteristics (Table 2). An average commute emission factor was used for all trials apart from the RESTART trial due to availability of primary data (Figure 3).

The total contribution from the six RCT’s to the carbon emissions of this module is 177,375 kgCO_2_e. This module was consistently the highest CO_2_e component for all of the RCT’s and within that the commute to the office accounted for >50% of the total CO_2_e for each RCT in this module. This makes it the largest overall contributing factor to the carbon footprint of each RCT.


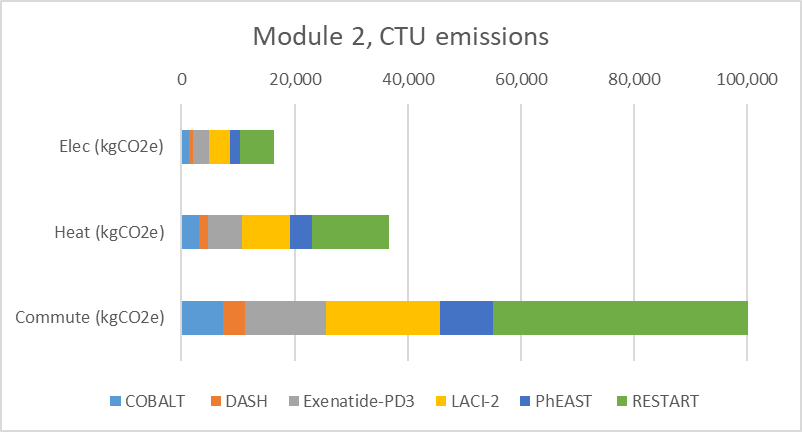


|  | Elec (kgCO2e) | Heat (kgCO2e) | Commute (kgCO2e) |
| --- | --- | --- | --- |
| COBALT | 1,377.27 | 3,074.87 | 7,323.00 |
| DASH | 731.64 | 1,633.45 | 3,890.22 |
| Exenatide-PD3 | 2,711.50 | 6,052.60 | 14,414.90 |
| LACI-2 | 3,769.35 | 8,415.42 | 20,042.10 |
| PhEAST | 1,762.90 | 3,935.80 | 9,373.54 |
| RESTART | 6,079.30 | 13,572.60 | 59,215.00 |

Supplementary Figure 2 Estimated sub components of module 2 for all RCT’s

Supplementary Figure 3 Estimated emissions for commuting in the RESTART trial

#### Module 3, Meetings & Travel

Module 3 (Figure 4) estimates the impact of meetings for the trials. For this project only SIVs, TSCs and DMCs are included. Information on study specific number of meetings, format (hybrid/in person) and travel details are presented in section 4.5.

The total contribution from the six RCT’s to the carbon emissions of this module is 5,906 kgCO_2_e and within that air travel accounted for the highest contribution to this module.


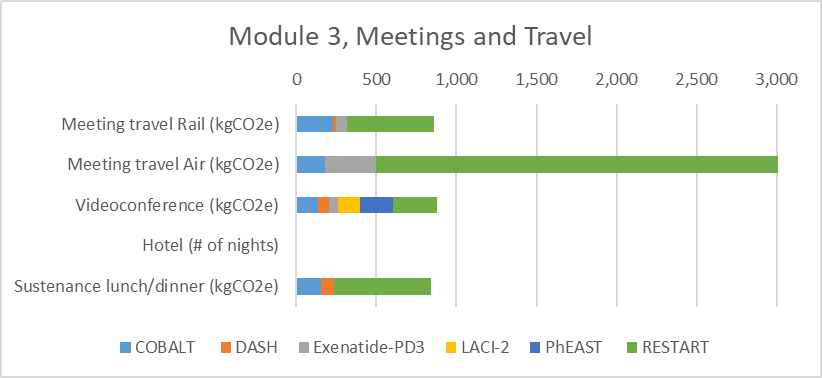


|  | Meeting travel Rail (kgCO2e) | Meeting travel Air (kgCO2e) | Videoconference (kgCO2e) | Hotel (# of nights) | Sustenance lunch/dinner (kgCO2e) |
| --- | --- | --- | --- | --- | --- |
| COBALT | 232.00 | 177.00 | 134.60 | N/A | 156.80 |
| DASH | 14.50 | N/A | 67.84 | N/A | 81.40 |
| Exenatide-PD3 | 70.40 | 318.00 | 58.70 | N/A | N/A |
| LACI-2 | N/A | N/A | 135.60 | N/A | N/A |
| PhEAST | N/A | N/A | 209.75 | N/A | N/A |
| RESTART | 544.31 | 2,829.93 | 271.29 | N/A | 603.84 |

Supplementary Figure 4 Estimated sub components of module 3 for all RCT’s

#### Module 4, Intervention

Module 4 (Figure 5) estimates the impact of the trial intervention. We were unable to calculate the emissions values of packaging as the data was not available. The emission factor for this section also does not include the manufacture of the IMP or device.

The total contribution from the six RCT’s to the carbon emissions of this module is 356 kgCO_2_e.


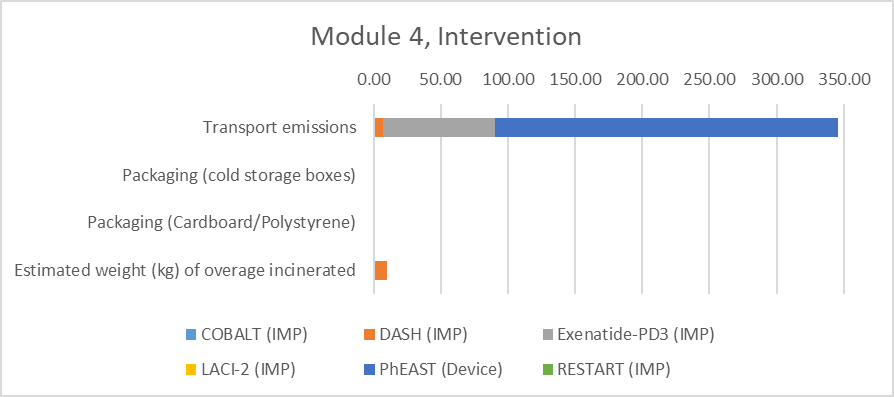


|  | Transport emissions | Packaging (cold storage boxes) | Packaging (Cardboard/Polystyrene) | Estimated weight (kg) of overage incinerated |
| --- | --- | --- | --- | --- |
| COBALT (IMP) | 1.10 | Unk | Unk | Unk |
| DASH (IMP) | 6.20 | Unk | Unk | 10.30 |
| Exenatide-PD3 (IMP) | 82.80 | Unk | Unk | Unk |
| LACI-2 (IMP) | N/A | N/A | N/A | N/A |
| PhEAST (Device) | 255.58 | N/A | Unk | N/A |
| RESTART (IMP) | N/A | N/A | N/A | N/A |

Supplementary Figure 5 Estimated sub components of module 4 for all RCT’s

#### Module 5, Data Collection & Exchange

Module 5 (Figure 6) estimates the impact of data collection and exchange during the course of a trial. Data was not available for three studies as they are still active and data exchange has not commenced. The number of emails sent for each trial is an estimate based on how many might be sent each day.

The total contribution from the six RCT’s to the carbon emissions of this module is 6,451 kgCO_2_e. Three studies are still active therefore they were not included in this component for end of study data exchange.

|  | Postage (data sent back to CTU) (kgCO2e) | CD manufacture for scans (kgCO2e) | Computer time to save scans (kgCO2e) | Emails (kgCO2e) | GB of data storage and transmission (kgCO2e) | Postage (data from patients sent to CTU) kgCO2e | Lab data sent to CTU. | Other collabs data to CTU eg Data linkage (kgCO2e) |
| --- | --- | --- | --- | --- | --- | --- | --- | --- |
| COBALT | N/A | N/A | N/A | 371.25 | Unk | N/A | N/A | N/A |
| DASH | 0.07 | 19.92 | 0.72 | 132.00 | 1.73 | N/A | N/A | N/A |
| Exenatide-PD3 | N/A | N/A | N/A | 1,089.00 | Unk | N/A | N/A | N/A |
| LACI-2 | 5.50 | 133.00 | 4.90 | 330.00 | Unk | 126.90 | N/A | N/A |
| PhEAST | N/A | N/A | N/A | 247.50 | Unk | N/A | N/A | 2,011.50 |
| RESTART | 37.50 | 488.00 | 17.72 | 1,125.00 | 9.66 | 545.89 | N/A | N/A |
| \|  \| \| --- \| |  |  |  |  |  |  |  |  |
|  |  |  |  |  |  |  |  |  |
|  |  |  |  |  |  |  |  |  |
|  |  |  |  |  |  |  |  |  |
|  |  |  |  |  |  |  |  |  |
|  |  |  |  |  |  |  |  |  |
|  |  |  |  |  |  |  |  |  |
|  |  |  |  |  |  |  |  |  |
|  |  |  |  |  |  |  |  |  |
|  |  |  |  |  |  |  |  |  |
|  |  |  |  |  |  |  |  |  |
|  |  |  |  |  |  |  |  |  |
|  |  |  |  |  |  |  |  |  |

Supplementary Figure 6 Estimated sub components of module 5 for all RCT’s

#### Module 6, Trial Supplies & Equipment

Module 6 (Figure 7) estimates the impact of supplies and equipment used by the trial team, for example, computers or laptops and equipment provided to the sites to carry out the trial assessments. This includes equipment for the participants. Some of the data was unavailable retrospectively, specifically details of a fridge supplied to one site. No other equipment was provided by the trials.

The total contribution from the six RCT’s to the carbon emissions of this module is 16,382 kgCO_2_e.


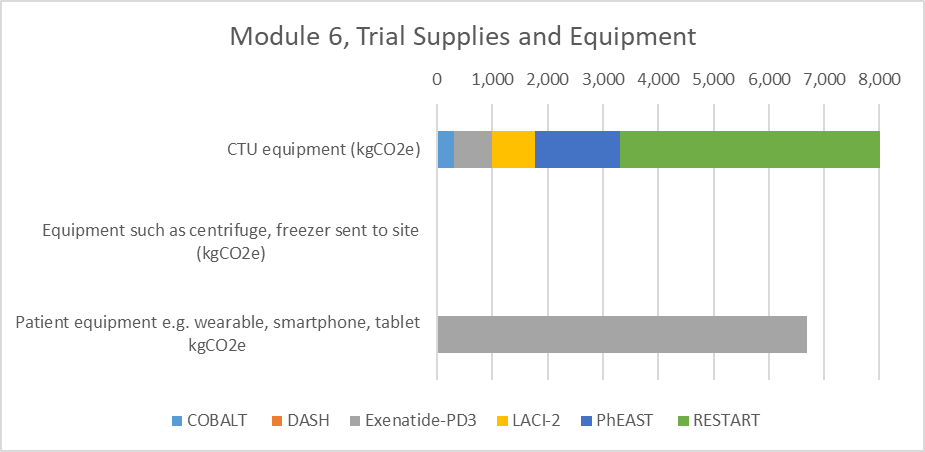


|  | CTU equipment (kgCO2e) | Equipment such as centrifuge, freezer sent to site (kgCO2e) | Patient equipment e.g. wearable, smartphone, tablet kgCO2e |
| --- | --- | --- | --- |
| COBALT | 301.00 | Unk | N/A |
| DASH | N/A | N/A | N/A |
| Exenatide-PD3 | 688.70 | Unk | 6,684.70 |
| LACI-2 | 774.00 | N/A | N/A |
| PhEAST | 1,548.00 | N/A | N/A |
| RESTART | 6,385.50 | N/A | N/A |

Supplementary Figure 7 Estimated sub components of module 6 for all RCT’s

#### Module 7, Trial Assessments

Module 7 (Figure 8) estimates the impact of trial assessments and includes the estimated carbon emissions associated with trial visits (in addition to standard care), blood samples, scans and time spent by local site staff on these assessments. Scans included MRI’s and CT’s, it did not include DaTSCAN’s for one study (Exenatide-PD3) as the emission factor was unavailable at the time. Blood samples included FBC, LFT, U & Es, coagulation tests and c-reactive tests. The emission factor for follow up telephone calls was also unavailable at the time.

The total contribution from the six RCT’s to the carbon emissions of this module is 58,457 kgCO_2_e. This module contributed the 2nd highest quantity of CO_2_e from all RCT’s, apart from the PhEAST trial which had no scans, study visits or sample collection apart from routine clinical care.

| \|  \| \| --- \| |  |  |  |  |  |
| --- | --- | --- | --- | --- | --- | --- |
|  |  |  |  |  |  |
|  |  |  |  |  |  |
|  |  |  |  |  |  |
|  |  |  |  |  |  |
|  |  |  |  |  |  |
|  |  |  |  |  |  |
|  |  |  |  |  |  |
|  |  |  |  |  |  |
|  |  |  |  |  |  |
|  | Patient travel (kgCO2e) | Other patient assessments: scans (kgCO2e) | Other patient assessments: bloods (kgCO2e) | Electricity consumption for trial staff at site (kgCO2e) | Heating for trial staff at site (kgCO2e) |
| COBALT | 6,102.00 | N/A | 32.25 | 1,313.64 | 2,468.52 |
| DASH | N/A | 993.60 | 9.77 | 33.50 | 62.90 |
| Exenatide-PD3 | 11,252.00 | N/A | 78.20 | 989.40 | 1,862.40 |
| LACI-2 | 8,421.60 | 8,966.10 | N/A | 1,002.00 | 1,792.10 |
| PhEAST | N/A | N/A | N/A | N/A | N/A |
| RESTART | 3,114.60 | 7,582.90 | N/A | 1,926.67 | 452.56 |

Supplementary Figure 8 Estimated sub components of module 7 for all RCT’s

#### Module 8, Sample Collection

#### &

#### Module 9, Laboratory

Module 8 (Figure 9) and module 9 (Figure 10) estimated carbon emissions associated with samples and laboratory use, they included shipping samples from site to the central lab, time spent by laboratory staff analysing the samples and subsequent storage of samples. Four of the six RCT’s (COBALT, LACI-2, PhEAST, RESTART) did not collect samples. The Exenatide-PD3 trial is currently active so an estimated CO_2_e component was not available for the sample analysis as the number of samples could not be confidently predicted. Details of the lab staff needed for analysis was also not available, therefore it was not included. For the DASH trial, these two modules did include the shipping materials for p selectin kits, and the production of dry ice and use of cold storage box for VWF/VIII samples shipped from sites to HQ. Laboratory staff time and storage of samples in a -80 freezer were also calculated for the DASH trial.

The total contribution from the six RCT’s to the carbon emissions of the sample’s module is 196 kgCO_2_e and to the laboratory module is 285 kgCO_2_e.

| \|  \| \| --- \| |  |  |  |  |
| --- | --- | --- | --- | --- | --- |
|  |  |  |  |  |
|  |  |  |  |  |
|  |  |  |  |  |
|  |  |  |  |  |
|  |  |  |  |  |
|  |  |  |  |  |
|  |  |  |  |  |
|  |  |  |  |  |
|  |  |  |  |  |
|  | Packs for sample collection (kgCO2e) | Sample kits shipped from supplier to CTU (kgCO2e) | Sample kits shipped from distributor to site (kgCO2e) | Samples shipped from site/patient to central lab (kgCO2e) |
| COBALT | N/A | N/A | N/A | N/A |
| DASH | 189.17 | N/A | 0.50 | 2.40 |
| Exenatide-PD3 | 4.34 | N/A | N/A | N/A |
| LACI-2 | N/A | N/A | N/A | N/A |
| PhEAST | N/A | N/A | N/A | N/A |
| RESTART | N/A | N/A | N/A | N/A |

Supplementary Figure 9 Estimated sub components of module 8 for all RCT’s

| \|  \| \| --- \| |  |  |  |  |
| --- | --- | --- | --- | --- | --- |
|  |  |  |  |  |
|  |  |  |  |  |
|  |  |  |  |  |
|  |  |  |  |  |
|  |  |  |  |  |
|  |  |  |  |  |
|  |  |  |  |  |
|  |  |  |  |  |
|  |  |  |  |  |
|  |  |  |  |  |
|  |  |  |  |  |
|  | Lab staff electricity (kgCO2e) | Lab staff heating (kgCO2e) | Processing and analysis of samples (kgCO2e) | Storage of samples e.g -80freezer (kgCO2e) |
| COBALT | N/A | N/A | N/A | N/A |
| DASH | 4.14 | 6.86 | N/A | 274.00 |
| Exenatide-PD3 | N/A | N/A | N/A | N/A |
| LACI-2 | N/A | N/A | N/A | N/A |
| PhEAST | N/A | N/A | N/A | N/A |
| RESTART | N/A | N/A | N/A | N/A |

Supplementary Figure 10 Estimated sub components of module 9 for all RCT’s

#### Module 10, Archiving & Trial Close Out

Module 10 (Figure 11) estimated carbon emissions associated with trial close out, this included components such as energy use of the storage unit (based on squared metre of floor space used for archive boxes) and return of any equipment on loan to sites when the trial is finished.

The total contribution from the carbon emissions of this module is 5,380 kgCO_2_e. The data for the three complete studies and for two of the three active studies was estimated in this module and the data was not available for one active study.

| \|  \| \| --- \| |  |  |  |  |  |
| --- | --- | --- | --- | --- | --- | --- |
|  |  |  |  |  |  |
|  |  |  |  |  |  |
|  |  |  |  |  |  |
|  |  |  |  |  |  |
|  |  |  |  |  |  |
|  |  |  |  |  |  |
|  |  |  |  |  |  |
|  |  |  |  |  |  |
|  |  |  |  |  |  |
|  |  |  |  |  |  |
|  |  |  |  |  |  |
|  |  |  |  |  |  |
|  |  |  |  |  |  |
|  |  |  |  |  |  |
|  | Archiving (files): Electrical (kgCO2e) | Archiving (files): Heating (kgCO2e) | Archiving (files): electronic data (kgCO2e) | Storage of any samples not included in other modules (kgCO2e) | Return of equipment from sites to CTU (kgCO2e) |
| COBALT | 1,006.40 | 1,808.80 | Unk | N/A | N/A |
| DASH | 211.34 | 379.84 | Unk | N/A | N/A |
| Exenatide-PD3 | 394.40 | 822.00 | Unk | N/A | N/A |
| LACI-2 | 75.48 | 135.70 | Unk | N/A | N/A |
| PhEAST | Unk | Unk | Unk | N/A | Unk |
| RESTART | 171.10 | 307.50 | 56.17 | N/A | N/A |

Supplementary Figure 11 Estimated sub components of module 10 for all RCT’s

# APPENDIX E: DETAILED BREAKDOWN OF RESULTS FOR INDIVIDUAL RCT’s

#### Results of the COBALT Trial

Supplementary Figure 12 Summary of the COBALT Trial estimated carbon footprint

Results from the COBALT trial are summarised in Figure 12. The COBALT trial is currently an active trial which intends to recruit 300 participants across 25 sites with a study duration of just over 3.75 years. The carbon footprint of the trial was estimated to be 26.3 tonnes (26,327.67 kgCO_2_e).

(i) Results show that trial team emissions accounted for 44.73% of the overall carbon footprint for this RCT. This is the largest estimated carbon footprint for this RCT (11,775.14 kgCO_2_e) with average commuting journeys responsible for 7,323 kgCO_2_e, heating costs were 3,074.9 kgCO_2_e and electricity costs were 1,3773.3 kgCO_2_e.

(ii) Patient assessments accounted for 37.67% of the overall carbon footprint for this RCT which was the second biggest estimated contributor to this trial (9,916.41 kgCO_2_e) with 3 study visits per participant (6,102 kgCO2e) as the main factor.

(iii) Archiving and trial close out accounted for 10.69% of the overall footprint for this RCT. This is the third biggest contributor (2,815.20 kgCO2e) and is based on 4 archive boxes per site and stored for 10 years.

(iv) Other estimated carbon emissions included:

- Trial specific meetings (2.66%, 700.40 kgCO2e). The majority of meetings, including SIV’s, were held online with a small number of in person meetings at trial headquarters.
- Trial set up (1.70%, 447.12 kgCO2e). This included the printing and transport of paper ISF’s for each site. Trial equipment (301 kgCO2e) included a small budget for trial team equipment (<£1,000).
- Data exchange (1.41%, 371.30 kgCO2e). Data collection and exchange included emails (371.3 kgCO_2_e) sent over the course of the study, it did not include the GB of the trial database which was unknown as the study is still active.
- Trial equipment (1.14%, 301 kgCO_2_e).

(v) Minimal estimated carbon emissions for this trial included shipment of the IMP for the trial at ambient temperature to each site (2.5 shipments per site) (1.1 kgCO2e).

(vi) There was no data linkage, scans or research sample collections in this trial.

#### Results of the DASH Trial

Supplementary Figure 13 Summary of the DASH Trial estimated carbon footprint

Results from the DASH trial are summarised in Figure 13. The DASH trial completed in June 2022 and recruited 54 participants with 10 sites and a study duration of just over 4 years. The carbon footprint of the trial was estimated to be 8.8 tonnes (8805.3 kgCO_2_e).

(i) Results show that trial team emissions accounted for 71.04% of the overall carbon footprint for this RCT. This is the largest estimated carbon footprint for this RCT (6,255.3 kgCO_2_e) with average commuting costs responsible for 3,890.2kgCO_2_e, heating costs were 1,633.5 kgCO_2_e and electricity costs were 731.6 kgCO_2_e.

(ii) Patient assessments accounted for 12.49% of the overall carbon footprint for this RCT which was the second biggest estimated contributor to this trial (1,099.8 kgCO_2_e) with 2 CT’s carried out for each participant (993.6 kgCO_2_e) and blood samples.

(iii) Archiving and trial close out accounted for 6.71% of the overall footprint for this RCT. This is the third biggest contributor (591.20 kgCO_2_e) and this is based on 3 archive boxes per site and stored for 7 years.

(iv) This RCT used a number of blood samples including P-selectin tests, coagulation tests and U&E’s. The carbon footprint of the laboratory staff (11 kgCO2e) for processing some of the samples, and the materials needed for the samples (3.24%, 189.2 kgCO2e) also contributed to the overall carbon footprint of the trial. Materials included distribution of sample kits to sites and transport of samples for analysis at the central laboratory.

(v) Other estimated carbon emissions included trial specific meetings (1.86%, 163.7 kgCO2e), including lunch provided and videoconferencing accounting for a small amount of the total. The SIV’s and DMC’s were all held remotely and half of the TSC’s (n=4) were hybrid and the other half (n=4) were remote.

(v) Minimal estimated carbon emissions for this trial inluded:

- Data collection and exchange (1.75%, 154.40 kgCO2e), this figure includes email exchange over the course of the study (132 kgCO_2_e) and shipment of scan CD’s from the site to trial HQ. The study did not have paperwork returned to trial HQ and there was no data linkage.
- Trial set up (0.54%, 47.3 kgCO2e) included the printing and transport of paper ISF’s for each of the 10 sites. Shipment of the refrigerated IMP for the trial to each site (varying between 1 - 4 shipments per site) also had an overall impact (0.19%, 6.2 kgCO2e).

(vi) There was no trial supplies or equipment data for this study.

#### Results of the Exenatide-PD3 Trial

Supplementary Figure 14 Summary of the Exenatide-PD3 Trial estimated carbon footprint

Results from the Exenatide-PD3 trial are summarised in Figure 14. The Exenatide-PD3 trial is currently an active trial in follow up phase which recruited 194 participants with 6 sites and a study duration of 5.5 years. The carbon footprint of the trial was estimated to be 47.6 tonnes (47,626kgCO_2_e).

(i) Results show that trial team emissions accounted for 48.67% of the overall carbon footprint for this RCT. This is the largest estimated carbon footprint for this RCT (23,179 kgCO_2_e) with average commuting costs responsible for 14,419.9 kgCO_2_e, heating costs were 6,052.6 kgCO_2_e and electricity costs were 2,711.5kgCO_2_e.

(ii) Patient assessments accounted for 29.78% of the overall carbon footprint for this RCT which was the second biggest estimated contributor to this trial (14,182 kgCO_2_e) with 10 study visits per participant (11,252 kgCO2e) as the main contributor. This study also had a number of blood samples collected (4.3kgCO2e). The carbon emissions of the DaTSCAN’s for a sub study were not included here as the carbon calculator tool does not yet have an emission factor available.

(iii) Trial supplies and equipment accounted for 15.48% of the overall footprint for this RCT. This is the third biggest contributor (7,373.4 kgCO2e) with the provision of a smart phone for remote monitoring (6,684.7 kgCO2e) as the main reason for a carbon footprint in this module.

(iv) Other estimated carbon emissions included:

- Archiving (2.55%, 1,216.40 kgCO2e)
- Data collection, (2.29%, 1,089 kgCO2e) including email exchange and meetings and travel (0.94%, 447.1 kgCO2e) with SIV’s held in person.

(v) Minimal estimated carbon emissions for this trial included shipment of the IMP for the trial to each of the six sites (25 shipments per site) (0.17%, 82.8 kgCO2e) and trial set up (0.11%, 447.1 kgCO2e)

(vi) There was insufficient information to calculate the estimated emissions related to laboratory staff and analysis. There were no questionnaires or diaries returned by post and there was no data linkage in this study.

#### Results of the LACI-2 Trial

Supplementary Figure 15 Summary of the LACI-2 Trial estimated carbon footprint

Results from the LACI-2 trial are summarised in Figure 15. The LACI-2 trial completed in December 2022 and it recruited 363 participants with 26 sites and a study duration of just over 5 years. The carbon footprint of the trial was estimated to be 54.92 tonnes (54,929 kgCO_2_e).

(i) Results show that trial team emissions accounted for 58.67% of the overall carbon footprint for this RCT. This is the largest estimated carbon footprint for this RCT (32,227 kgCO_2_e) with average commuting costs responsible for 20,042.10 kgCO_2_e, heating costs were 8,415.40 kgCO_2_e and electricity costs were 3,769.40 kgCO_2_e.

(ii) Patient assessments accounted for 36.74% of the overall carbon footprint for this RCT which was the second biggest estimated contributor to this trial (20,182 kgCO_2_e) with one MRI per participant (n=363) (8,966.10 kgCO2e) and 4 study visits (8,421.60 kgCO2e) as the main contributors.

(iii) Trial set up accounted for 1.46% of the overall footprint for this RCT. This is the third biggest contributor with the transporting of ISF’s to 26 sites as the main source of emissions (799.29 kgCO_2_e).

(iv) Other estimated carbon emissions included;

- trial equipment (1.41%, 774 kgCO2e) for data linkage costs
- Data collection and exchange (1.09%, 600.30 kgCO2e) for the return of follow up questionnaires and scan CD’s.

(v) Minimal estimated carbon emissions for this trial included the archiving of paperwork and scan CD’s (0.38%, 211.18 kgCO_2_e) and online trial specific meetings and travel (0.25%, 135.60 kgCO_2_e). The majority of meetings, including SIV’s, were held online with a small number of in person meetings at trial headquarters.

(vi) There was no impact on carbon emissions from shipment of the intervention as the IMP used is prescribed locally as part of standard practice. There were no sample collections in the trial.

#### Results of the PhEAST Trial

Supplementary Figure 16 Summary of the PhEAST Trial estimated carbon footprint

Results from the PhEAST trial are summarised in Figure 16. The PhEAST trial is currently an actively recruiting trial which intends to recruit 800 participants with 50 sites planned and an anticipated study duration of just over 3 years. The carbon footprint of the trial was estimated to be 19.1 tonnes (19,098 kgCO_2_e).

(i) Results show that trial team emissions accounted for 78.92% of the overall carbon footprint for this RCT. This is the largest estimated carbon footprint for this RCT (15,072 kgCO_2_e) with average commuting costs responsible for 9,373.50 kgCO_2_e, heating costs were 3,935.8 kgCO_2_e and electricity costs were 1,762.90 kgCO_2_e.

(ii) Data collection and exchange accounted for 10.54% of the overall carbon footprint for this RCT which was the second biggest estimated contributor to this trial (2,012.60 kgCO_2_e) with intended data linkage as the biggest contributor to the carbon footprint (1,548 kgCO_2_e). Email exchange for the duration of the trial is also included (247.50 kgCO_2_e). It was not possible to quantify the impact of follow up questionnaires or storage size (gigabytes) of the trial database as the study is still in early stages and data was not available.

(iii) Trial equipment accounted for 8.11% of the overall footprint for this RCT. This is the third biggest contributor (1,548 kgCO_2_e) with <£2,000 budget used for equipment.

(iv) Other estimated carbon emissions included:

- The distribution and return of the intervention (PES base and catheters) to each site (1.34%, 255.58 kgCO_2_e)
- Remote trial specific meetings and travel (1.10%, 209.75 kgCO_2_e).

(v) The estimated carbon emissions for trial set-up had minimal impact (0.03 kgCO_2_e) due to the use of electronic investigator site files (eISF’s).

(vi) There was no additional patient assessments or study visits as the participants were already admitted to hospital when recruited with follow up assessments as part of routine clinical care so there was no associated carbon emissions, and there was no sample collections in the trial. It was not possible to quantify the archiving footprint as the study is currently active.

#### Results of the RESTART Trial

Supplementary Figure 17 Summary of the RESTART Trial estimated carbon footprint

Results of the RESTART trial are summarised in Figure 17. The RESTART trial completed in 2021, recruited 537 participants across 112 sites with a study duration of 8 years. The carbon footprint of the trial was estimated to be 106.40 tonnes (106,429.13 kgCO_2_e).

(i) Results show that trial team emissions accounted for 74.10% of the overall carbon footprint for this RCT. This is the largest estimated carbon footprint for this RCT (78,866.53 kgCO_2_e), with estimated commuting costs responsible for 59,214.6 kgCO_2_e, heating costs were 13,572.6 kgCO_2_e and electricity costs were 6,079.3 kgCO_2_e.

More detailed data was available for this particular trial and it was therefore possible to calculate specific commute costs for each mode of transport (Figure 18), unlike other trials where specific details were not available and an average commute factor was applied.

Supplementary Figure 18 Breakdown of commute emissions for the duration of the RESTART Trial

(ii) Patient assessments accounted for 12.29% of the overall carbon footprint for this RCT which was the second biggest estimated contributor to this trial (13,076.70 kgCO_2_e) and 7,582.9 kgCO_2_e of this was a result of the use of MRI scans (n=307).

(iii) Trial supplies and equipment accounted for 6.00% of the overall footprint for this RCT. This is the third biggest contributor (6,385.50 kgCO_2_e) with the estimated data linkage process (6,385.50 kgCO_2_e) as the main source of emissions.

(iv) Other estimated emission sources included:

- Trial specific meetings and travel (3.99%, 4,249.40 kgCO_2_e). All SIVs and DMC meetings were held remotely, however TSC meetings (n=17 meetings) for the duration of the study were hybrid with some of the committee travelling by domestic air/train.
- Data collection and exchange also resulted in an estimated carbon emission source (2.09%, 2,223.80 kgCO_2_e) and this included the return of standard care CT and MRI scans via either electronic upload or CD postage plus an annual follow up questionnaire from GP’s and the participant.

(v) Minimal estimated emissions for this trial included trial set up (1.03%, 1092.40 kgCO_2_e) close out and archiving for 122 sites (0.50%, 534.80 kgCO_2_e). There was no sample collection in the RESTART study so there was no impact from samples or laboratory emissions. There was no impact on carbon emissions from shipment of the intervention as the IMP used is prescribed locally as part of standard practice.

# Appendix F: Characteristics of clinical trials for neurological disorders included in this study

#### Table 2, Characteristics of clinical trials included in this study

Active = Trial still active at the time of data collection. FTE = full time equivalent staff members. CTIMP = clinical trial of an investigational medicinal product. CT = computed tomography. MRI = magnetic resonance imaging. A detailed breakdown of each RCT can be found in Appendix E, supplementary materials.

(see Appendix F, supplementary file)


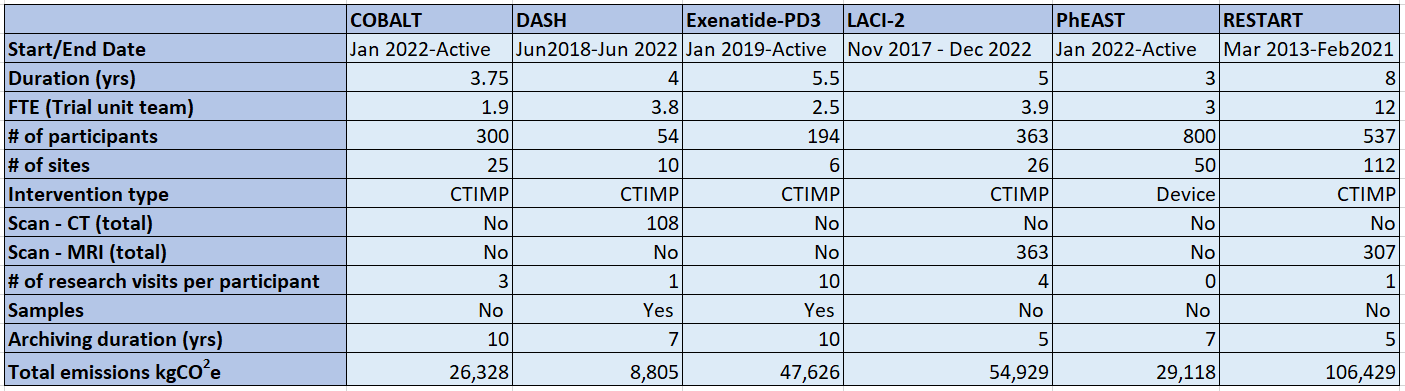


# Appendix G: Relative contributions of each module to the carbon footprint of each clinical trial

#### Table 3, Total estimated CO^2^e per RCT with proportional estimated contribution per module
