# Supplementary material for: Carbon footprint of a sample of clinical trials for people with neurological disorders: cross-sectional analysis
Source: BMJ Open. 2025 Jun 17;15(6):e090419. doi: 10.1136/bmjopen-2024-090419 (PMC12182044; doi:10.1136/bmjopen-2024-090419)
Supplement: Supplementary file 2 [file bmjopen-2024-090419.reviewer_comments.pdf]

PEER REVIEW HISTORY

BMJ Open publishes all reviews undertaken for accepted manuscripts. Reviewers are asked to complete a checklist review form and are provided with free text boxes to elaborate on their assessment. These free text comments are reproduced below.

ARTICLE DETAILS

Title (Provisional)

The carbon footprint of a sample of clinical trials for people with neurological disorders: cross-sectional analysis

Authors

Cranley, Denise; Dunn, Sarah; Craig, Jennifer; McComish, Rachel; Heye, Anna; Innes, Karen; Dinsmore, Lynn; Griffiths, Jessica; Fox, Lisa; Al-Shahi Salman, Rustam; Taylor, John-Paul; Desborough, Michael JR; Sprigg, Nikola; Foltynie, Thomas; Wardlaw, Joanna; Oatey, Katherine; Bath, Philip; Williamson, Paula R

VERSION 1 - REVIEW

**Reviewer**	**1**
**Name**	**Bronstein, Jeff**
**Affiliation**	**University of California Los Angeles, Neurology**
**Date**	**26-Aug-2024**
**COI**	**None**

This study addresses an important topic that is rarely discussed in my experiences with clinical trials; The carbon footprint. Cranley et al. determined the carbon footprint of 6 clinical trials for a variety of neurological conditions in the UK after performing a systematic search of the 2 main databases. They found that emissions by clinical trials staff produced the largest carbon footprint by far relative to several other trial activities. They provided a detailed analysis for each study as well as summary data which will be useful for reducing the footprint for future trials. This is a novel study in neurology and 1 of only a few I am aware of studying clinical trial footprint in general.

The authors describe the strengths and weaknesses of their study fairly well but 1 consideration was not mentioned. Some of the activities such as travel of the staff to the study site or to an investigators' meeting may serve more than 1 purpose so the actual carbon contribution might be lower in reality. For example, many investigator meeting occur at a national or international congress and the participants would be traveling there regardless of the study. Clinical trial coordinators likely have to go to the trial site for many

activities so attributing all of the carbon to their travels in not really accurate. I understand it would be difficult to account for these other activities but the issue should be discussed.

Reviewer 2

Name Ó Breasail, Mícheál

Affiliation Population Health Sciences, Bristol Medical School, University of Bristol, 1-5 Whiteladies Road, Bristol BS8 1NU, UK

Date 18-Nov-2024

COI NA

This is a clearly written and interesting manuscript which addresses the important and much overlooked impact of clinical research on carbon emissions.

However, based on the limited number of studies included it is difficult to discern whether these 6 studies provide an accurate representation of the wider neurological field. Some additional detail may help contextualise the findings for the reader.

In particular further expansion on how representative these trials are of others within their respective subdisciplines (say PD vs stroke) would be welcome e.g. are trials discussed typical of the field in terms of size (patients and n of sites), duration and complexity(number of visits, length of follow up)?

Equally the decision to include trials over the preceding 5 years has the unfortunate effect of covering the COVID-19 pandemic, as acknowledged in the limitations changes to protocols to mitigate the pandemic are not accounted for, as such I think the justification for selecting this retrospective period needs to be provided more explicitly in the introduction/methods and discussion.

Finally there are a couple of references that don't seem to have rendered correctly on pages 11-12.

Reviewer 3

Name LaRoche, Jason

Affiliation Janssen Research and Development LLC, Janssen Clinical Innovation

Date 17-Mar-2025

COI I have no competing interests to declare.

Dear Dr. Denise Cranley,

Thank you for submitting your valuable research as it contributes to a growing body of knowledge around the climate impact of clinical trials. I have reviewed your manuscript and added comments in the attached PDF, but also list my comments as follows:

- Page-4, line-37, add a citation to your statement about neurological disorders being a leading cause of disease burden. The citation appears in the body of your manuscript and should be pulled forward.

- Page-5, line-67, you should define the abbreviation “RCTs”. I believe you first make reference to randomized control trials in the prior Methods section. Introduce the abbreviation there.

- Page-5, line-70, add a citation for your reference to the CRASH trials. If I recall correctly, there were two publications based upon the original CRASH1 and CRASH2 studies.

- Page-5, line-72, add a citation for the guidance documents from the Low Carbon Clinical Trials Group. The details here may be sufficient to find the document in an internet search but you should be more specific and provide a link to the landing page or other details for clarity.

- Page-7, line-100, you mention that the three trials from Astra Zeneca used an in-house carbon calculator tool. Was the output output of this tool used in your analysis? If so, how did the calculation/methodology of this tool compare with the methodology of the Low Carbon Clinical Trials Group? Was it the same or different? If different, how would this impact your results? This should be covered in the discussion.

- Page-11, line-188, why were only 9 of 318 trials found to be eligible? You mentioned your selection criteria focused on UK-led and UK-recruiting trials. Did only 9 meet this criteria? I found the difference to be significant and as a reader I want to know what was the driving criteria behind such a low eligibility.

- Page-14, line-262, the overall phrase “commute accounting for the <50% of each RCT” reads very awkwardly and I would suggest revising. Saying that it accounts for "<50%" is a very broad statement as anything with a contribution of 0-49% would be included. At the same time, you're saying it's very significant. Perhaps say instead that it "accounts for no less than X and no more than Y percentage of emissions".

- Page-14, line-268, did you include emissions associated with the manufacture of the intervention or are you referring only to the emissions from sourcing the intervention? You should be more specific as this is unclear. Other published research that examined the emissions of drug product manufacture and packaging found it to have a very significant impact on overall trial emissions. If you’re including emissions from drug product manufacture and packaging then this is statement is much more powerful than if it does not.

- Page-17, line-330, From my own experience, this statement may be a bit risky. In my experience, cloud-based software solutions like telemedicine have a low footprint. However, hardware can have a very significant footprint. If for example, you need to provision a

smartphone to patients so they can use telemedicine capabilities then you're introducing roughly 56kg of CO2e for an iPhone 8. If you provision smartphones to 20 patients then you'll introduce more emissions than what you reported for "trial supplies and equipment" in your results. It might be a valuable to add a caveat that technology-use is favored when it utilizes existing hardware in the hands of sites and patients but caution against the introduction of new hardware.

Thank you again for submitting this valuable research and I look forward to seeing your revisions.

Kind Regards,

Jason LaRoche

**** The reviewer provided a marked copy with additional comments. Please contact the publisher for full details. ****

VERSION 1 - AUTHOR RESPONSE

Reviewer: 1
Dr. Jeff Bronstein, University of California Los Angeles Comments
to the Author:
This study addresses an important topic that is rarely discussed in my experiences with clinical trials; The carbon footprint. Cranley et al. determined the carbon footprint of 6 clinical trials for a variety of neurological conditions in the UK after performing a systematic search of the 2 main databases. They found that emissions by clinical trials staff produced the largest carbon footprint by far relative to several other trial activities. They provided a detailed analysis for each study as well as summary data which will be useful for reducing the footprint for future trials. This is a novel study in neurology and 1 of only a few I am aware of studying clinical trial footprint in general.

Response: We thank Dr Bronstein for these comments.

The authors describe the strengths and weaknesses of their study fairly well but 1 consideration was not mentioned. Some of the activities such as travel of the staff to the study site or to an investigators' meeting may serve more than 1 purpose so the actual carbon contribution might be lower in reality. For example, many investigator meeting occur at a national or international congress and the participants would be traveling there regardless of the study. Clinical trial coordinators likely have to go to the trial site for many activities so attributing all of the carbon to their travels in not really accurate. I understand it would be difficult to account for these other activities but the issue should be discussed.

Response: We would like to thank Dr Bronstein for this insight, and for taking the time to review and comment on the manuscript. We agree with this point. We also

agree that it is difficult to account for these nuances in detail, and we do refer to the carbon footprint as estimates. We have now added a clarification to the discussion: *Row 336 – 338: Additionally, we have not accounted for multi-purpose meetings and trips and there may be efficiency gains from travelling to some meetings that serve multiple purposes.*

Reviewer: 2
Dr. Mícheál Ó Breasail, Population Health Sciences, Bristol Medical School, University of Bristol, 1-5 Whiteladies Road, Bristol BS8 1NU, UK Comments to the Author:
This is a clearly written and interesting manuscript which addresses the important and much overlooked impact of clinical research on carbon emissions.

Response: We thank Dr Ó Breasail for these comments.

However, based on the limited number of studies included it is difficult to discern whether these 6 studies provide an accurate representation of the wider neurological field. Some additional detail may help contextualise the findings for the reader.

In particular further expansion on how representative these trials are of others within their respective subdisciplines (say PD vs stroke) would be welcome e.g. are trials discussed typical of the field in terms of size (patients and n of sites), duration and complexity(number of visits, length of follow up)?

Response: We would like to thank Dr Ó Breasail for taking the time to review our manuscript and for highlighting this issue regarding generalisability of the findings. Firstly, we have addressed this in the Strengths and Limitations section. We added a sentence to the discussion (row 310 – 317) stating, "*We were able to include twothirds of the eligible clinical trials, but the generalisability of six clinical trials about dementia, movement disorders, and stroke to all neurological disorders is uncertain*. *Similarly, this study included both drug and a device trial, with a range of trial outcomes*."

We acknowledge it would have been interesting to consider it in more depth. We had invited 3 additional studies to take part which could have broadened the field further (a multiple sclerosis study, an Alzheimer's study and a meningitis study) but we were unable to progress with data collection due to circumstances out of our control. Additionally, the results from the registries were naturally weighted towards neurovascular disorders (e.g. stroke) due to the number of research studies in that field. In rows 322 to 323 we also state, "However, only a small number of RCTs were eligible and included in this study and the six RCT designs were heterogenous." Additionally, in the Recommendations for Future Research section we also refer to the need to include a broader and larger sample size to generalise findings across neurology and state in rows 344-345, "Future research should enlarge our sample size and study a broader selection of RCTs for neurological disorders."

Equally the decision to include trials over the preceding 5 years has the unfortunate effect of covering the COVID-19 pandemic, as acknowledged in the limitations changes to protocols to mitigate the pandemic are not accounted for, as such I think the justification for selecting this retrospective period needs to be provided more explicitly in the introduction/methods and discussion.

Response: Thank you for this insight, we have now added an additional justification (Methods, row 150 – 152, “*Trials were included if they were registered within the previous 5 years, to ensure the results are relevant to current practice and to ensure we reflected the carbon footprint of contemporary trial designs.”* It can be difficult to acknowledge all the complexities of this subject due to word limitations but we can take the opportunity to explain a little further. Clinical research design is an evolving environment and trial design changes over time, for example, with the development of adaptive multi-platform trials. The objective for using a 5-year time period for the inclusion criteria was to ensure we included carbon footprint measurements of some elements of recent trial designs to make the results more relatable for carbon emissions. Equally, there are many neurology disorder trials registered on databases and we needed to be able to narrow these down with a reasonable timepoint criteria, hence the 5-year timepoint. The study was developed in Autumn 2023 so a post-Covid time window will have been too restrictive.

We agree that it would also be very interesting to complete a piece of research, similar to the Nightlife study (Quann et. al, 2023) which is referenced, which examines how a number of trial protocols had to adapt to the pandemic and the impact on the carbon footprint of those trials. Unfortunately for our study we had to focus on the protocol version in place at the time of data collection and take that snapshot of the trial. It was beyond the scope of our resources to consider how the protocol had changed, which, as the reviewer has stated, is acknowledged in the limitations. However, we have now clearly stated this in the manuscript, row 346348: *Additionally, future examination of trial protocol adaption as a result of COVID 19 could provide important insights into which factors might drive carbon footprint changes*.

These are both very interesting questions but unfortunately, we had to focus our calculations on one aspect for this study.

Finally there are a couple of references that don't seem to have rendered correctly on pages 11-12.

Response: We are grateful to Dr Ó Breasail for noticing this, both were related to Table 2 and we have updated it.

Reviewer: 3 (see also attached)
Dr. Jason LaRoche, Janssen Research and Development LLC, Delft University of Technology
Comments to the Author:

Dear Dr. Denise Cranley,

Thank you for submitting your valuable research as it contributes to a growing body of knowledge around the climate impact of clinical trials. I have reviewed your manuscript and added comments in the attached PDF, but also list my comments as follows:

Response: We thank Dr LaRoche for these comments.

Row 37 & 38: Neurological disorders were the leading cause of overall disease burden globally in 2021.
Can you find a citation for this statement? What is the basis of measure? Is it incidence rate across patient populations, resource (e.g. financial) consumption?

Response: This statement is based on the conclusions and figures available from the 2021 Global Burden of Disease (GBD) study which found the 37 conditions affecting the nervous system were collectively ranked as the leading group cause of DALYs in 2021 (443 million, 95% UI 378-521), affecting 3·40 billion (3·20-3·62) individuals (43·1%, 40·5-45·9 of the global population); global DALY counts attributed to these conditions increased by 18·2% (8·7-26·7) between 1990 and 2021. We have referenced the relevant GBD studies (11 and 15).

Row 48: We found 318 trials, nine were eligible…
Why so few? Was it because only 9 met your criteria of being UK-led and recruiting? I would hope to find the answer in the body of the document. I think it's ok to leave the question lingering in the abstract as it does hook me, as a reader, in and make me want to read the rest to learn the answer.

Response: We appreciate this opportunity to explain our rationale for this method. Due to time and resource limitations of this dissertation we had made the decision to keep the number of studies low (<10). A large number of results remained in the search results spreadsheet (n=150) when we began to apply the final exclusion criteria of 'no protocol available to us online' and it was not possible to search all of these results for availability of a protocol. Therefore, the decision was taken to search the results of each condition that remained in the search results spreadsheet in ascending numerical order until a study with an available protocol was located. The remaining search results were not checked as we had confirmed a result which had an available protocol for that study. The other criteria with the highest number of results included (i) removing duplicates from the results list (n=43) and (ii) studies which were not UK sponsored (n=37).

Row 67: RCTs
You should define the abbreviation "RCT" as it may be unfamiliar to some readers. I believe you first refer3ence randomized control trials in the Methods section so perhaps define it there?

Response: We have now added the “RCT” abbreviation to row 42 (Methods).

Row 70: CRASH trials
May want to add a citation here.

Response: We have now added the CRASH trials citation to Strengths and Limitations.

Row 72, 73 and 74: Low Carbon Clinical Trials 73 Group “Detailed Guidance and method to calculate the carbon footprint of a clinical 74 trial”.
Add a further citation to help the reader find it.

Response: We have now added a further citation for this statement.

Row 100 & 101: three trials funded by AstraZeneca using an in-house carbon calculator tool
Did you rely on the output of this in-house carbon calculator tool in your analysis? How did the calculations/methodology behind this in-house tool differ (if at all) from the methodology of the iLCCT guidance used in the other studies? This should be addressed in the discussion.

Response: We did not rely on the output of this Astra Zeneca in-house carbon calculator tool in our analysis, we used the summary of findings in their publication. The carbon calculator tool which the Astra Zeneca team used is not available for public use. Upon examination of the supplemental material for their publication we were able to determine that their carbon calculator tool was based on the Greenhouse Gas protocols (www.ghg.com) which also forms the basis for part of the NIHR-funded Detailed Guidance and method which we used to calculate the carbon footprint of our clinical trials for this study. The pharmaceutical industry and publicly funded, academic trialists are collaborating in the area of lower carbon clinical trials through the industry Low Carbon Clinical Trials Group (iLCCT) convened via the Sustainable Markets Initiative and supported by the Sustainable Healthcare Coalition and the MRC NIHR Trials Methodology Research Partnership Greener Trials groups. These groups have worked together to develop a common methodology framework for the assessment of environmental impact of clinical trials, and guidance exists for both sectors within that common method framework.

Row 188: nine were eligible for this study
Why were only 9 of the 318 eligible, was it because only 9 of the 318 were UK-led and recruiting? What was the driving criteria that resulted in so few being eligible?

Response: We appreciate this opportunity to explain our rationale. Due to time and resource limitations of this dissertation we had made the decision to keep the number of studies low (<10). A large number of results remained in the search results spreadsheet (n=150) when we began to apply the final exclusion criteria of ‘no protocol available to us online’. It was not possible to search all of these results for

availability of a protocol. Therefore, the decision was taken to search the results of each condition that remained in the spreadsheet in ascending numerical order until a study with an available protocol was located. The remaining search results were not checked as we had confirmed a result which had an available protocol for that study. The other criteria with the highest number of results included (i) removing duplicates from the results list (n=43) and (ii) studies which were not UK sponsored (n=37).

Row 262: for the <50% of each RCT
Consider rephrasing as I'm not sure what you're trying to say here. Saying that it accounts for "<50%" is a very broad statement as anything with a contribution of 0-49% counts. At the same time, you're saying it's very significant. Perhaps say instead that it "accounts for no less than X and no more than Y percentage of emissions".

Response: We are grateful to the reviewer for commenting on this. This is an error and it should have read '>50%'. It is correct to say that the commute of the trial team is significant as it contributes to greater than 50% of the emissions for each trial team module. The range is 62% to 75%, so we have used this instead. Page 36 of the supplemental material goes into more detail of this module.

Row 268: RCT than an intervention which did not require temperature control
Did you include emissions associated with the manufacture of the intervention or are you referring only to the emissions from sourcing the intervention?

Response: We did not include the emissions associated with the manufacture of the intervention as the emission factors for this are beyond the scope of the carbon calculator tool and are not available. The supplemental material goes into more detail about the specifics of the modules. Page 38, row 19 of the supplemental material includes a statement that the manufacture of the IMP is not included in this module. We have now added clarification to row 275: (*the emissions associated with the manufacture of the IMP were beyond the scope of this tool and were not included*).

Row 330 & 331: Trial teams should consider maximising the use of technology ***From my own experience, this statement may be a bit risky. In my experience, cloudbased software solutions like telemedicine have a low footprint. However, hardware can have a very significant footprint. If for example, you need to provision a smartphone to patients so they can use telemedicine capabilities then you're introducing roughly 56kg of CO2e for an iPhone 8. If you provision smartphones to 20 patients then you'll introduce more emissions than what you reported for "trial supplies and equipment" in your results. It might be a valuable to add a caveat that technology-use is favored when it utilizes existing hardware in the hands of sites and patients but caution against the introduction of new hardware.***

Response: We thank the reviewer for this insight and we agree with the point made. The carbon footprint of additional hardware (e.g. smartphones) for a trial is considered and

captured in more detail in Module 5 of the carbon calculator tool. We have now added a sentence to clarify our statement: “Preference should be given to using existing technology and hardware to minimise the carbon footprint of trial equipment, e.g., avoid the provision of new smartphones unless absolutely necessary.”

VERSION 2 - REVIEW

Reviewer 3

Name LaRoche, Jason

Affiliation Janssen Research and Development LLC, Janssen Clinical Innovation

Date 29-Apr-2025

COI

Dear Dr. Denise Cranley,

Thank you for submitting your revisions to this valuable manuscript. I have reviewed the document and I am satisfied with your responses to my previous questions and concerns. My only remaining comments are minor ones:

1) In line 264 you reference “CTU” for the first time and some readers may be unfamiliar with this abbreviation. I recommend spelling out the full word in the first reference and providing the abbreviation, example: “clinical trial unit (CTU)”.

2) In line 316 you introduce the colloquial term "-80 freezer" and reference it again in lines 425-426 where you discuss the opportunity to reduce emissions by using a "-70 freezer" instead. With the introduction of a comparison, I feel this warrants adding the unit of measure (C) to the reference for clarity.

Thank you again for submitting this valuable contribution to our understanding of the environmental impact of clinical research.

Kind Regards,

Jason

VERSION 2 - AUTHOR RESPONSE

Reviewer 3 had an additional minor revision which was related to the use of the CTU abbreviation. This has now been amended.

Reviewer 3 had a further additional minor revision which was related to the absence of a

unit of measurement for the freezer temperatures. This has now been updated in the manuscript.
